# The effects of humic substances on DNA isolation from soils

**DOI:** 10.7717/peerj.9378

**Published:** 2020-07-24

**Authors:** Ewa Wnuk, Adam Waśko, Anna Walkiewicz, Piotr Bartmiński, Romualda Bejger, Lilla Mielnik, Andrzej Bieganowski

**Affiliations:** 1 Institute of Agrophysics, Polish Academy of Sciences, Lublin, Poland; 2 Faculty of Food Science and Biotechnology, Department of Biotechnology, Microbiology and Human Nutrition, University of Life Sciences, Lublin, Poland; 3 Department of Geology, Soil Science and Geoinformation, Maria Curie-Skłodowska University, Lublin, Poland; 4 Department of Bioengineering, West Pomeranian University of Technology, Szczecin, Poland

**Keywords:** Humic acids, Fulvic acids, Soil use, DNA extraction

## Abstract

**Background:**

Humic substances (HS) are compounds with a complicated structure, present in the humus soil layer, water, lake sediments, peat, brown coal and shales. Due to their similar physicochemical properties to DNA, they may have an adverse effect on the subsequent use of the isolated material. The main aim of this research was to examine the effect of HS on DNA isolation depending on the soil type and land use, taking into account the spectroscopic full characteristics of HS fractions.

**Methods:**

The research was conducted on eight types of soil sample. Soils represented the most important Soil Reference Groups for temperate climates: Fluvisols, Regosols, Cambisols, Arenosols, Histosols and Luvisols. Soil samples were also collected from areas diversified in terms of use: arable land, grassland and forest. The extraction of HS fractions was performed using the procedure recommended by the International HS Society. The fractional composition of HS was characterized by UV–Vis and fluorescence methods. Soil DNA is extracted by direct cell lysis in the using a CTAB-based method with a commonly-used commercial soil DNA isolation kit. The basis for assessing the quantity and quality of extracted DNA was the Polymerase chain reaction (PCR) reaction since the analysis of soil DNA often relies on the use of PCR to study soil microorganisms.

**Results:**

Based on the results, it can be concluded that in the presence of a high concentration of HS, the isolated DNA was low quality and the additional purification procedure was necessary. Despite the differentiation of the internal structure of HS fractions, the decisive factor in the efficiency of DNA isolation from soil samples was the total carbon content in HS. Reduced DNA yields can significantly constrain PCR detection limits to levels inadequate for metagenomic analysis, especially from humus-rich soils.

## Introduction

Humic substances (HS) are one of the most important soil components (Weber et al., 2018). As polymer substances with complex structures, they are formed by the condensation of the biomass of microorganisms, plants and animals (Hayes & Clapp, 2001; Niemiałkowska-Butrym et al., 2012; Orsi, 2014; Śliwinska & Drab, 2015). Due to their heterogeneous nature, HS are characterized as a ‘three-dimensional phase’ (Stevenson, 1994) with the ability to bind other substances including minerals (Petridis et al., 2014), ions (Boguta & Sokołowska, 2016), contaminants (Ukalska-Jaruga, Smreczak & Klimkowicz-Pawlas, 2018) and water (Orsi, 2014) into reactive functional groups.

There are three fractions of HS: humic acids (HA) which are soluble at high pH values, fulvic acids (FA) which are soluble under all pH conditions and humin which is insoluble under all pH conditions (Ishiwatari, 1992). From a chemical point of view, HS are heteropolycondensates or complex polyanions (Stevenson, 1994). However, from a physical point of view, they are polydisperse substances (Matuszak-Slamani et al., 2017; Bejger et al., 2018; Ukalska-Jaruga, Debaene & Smreczak, 2018), with a wide range of molecular structure sizes. The HS have a range of specific chemical and physical properties, such as: a high sorption capacity (Ukalska-Jaruga et al., 2015; Ćwieląg-Piasecka et al., 2018), and high energy absorption in the whole spectrum range, especially in the UV and IR radiation ranges of the electromagnetic spectrum (Fooken & Liebezeit, 2003; Gołębiowska et al., 2005; Mielnik & Kowalczuk, 2018; Rodríguez, Schlenger & García-valverde, 2016; Shin, Monsallier & Choppin, 1999). They are also capable of transferring energy between molecules, which makes them very efficient light-sensitive compounds (Gieguzynska et al., 2009; Nkhili et al., 2014). Due to the bipolarity of HS (Winterwerp & Van Kesteren, 2004) they are responsible for creating a granule-like soil structure and can influence (similarly to detergents) the surface tension and viscosity of water.

The DNA analysis of soils is becoming increasingly popular (Verma, Singh & Sharma, 2017). This is unsurprising, as a very high biodiversity is present in soil (Wolińska et al., 2017; Frąc et al., 2018) and such analysis enables information to be obtained about present and past soil flora and microorganisms and helps in monitoring the soil processes (Torsvik & Ovreås, 2002; Chen et al., 2007; Yoccoz et al., 2012; Szafranek-Nakonieczna et al., 2018). However, the final result (i.e., the information about the soil processes) based on the DNA analysis must be preceded by a DNA isolation process. The isolation of DNA from the soil should provide a pure mixture of HS that would enable further steps of DNA analysis. However, a problem can occur in DNA analysis. The humic acids in soil have similar size and charge characteristics to DNA resulting in their co-extraction. This can influence the efficiency of the process and the purity of the DNA (Tebbe & Vahjen, 1993) and can cause difficulties in DNA isolation (Wilson, 1997; Hu et al., 2010). This difficulty has been noted in DNA purification procedures (Young et al., 1993; Sharma, Capalash & Kaur, 2007). However the interference of HS and DNA in soil was investigated in single soils. Systematic studies of soils with different origins and HS characterization have not been conducted.

The aim of this study was to evaluate the fractional composition of HS and its influence on DNA isolation processes in different soil types under different land usage.

## Materials and Methods

### Soil sampling

Analyses were performed on eight soil samples, collected from the humus horizon (0–15 to 0–30 cm, depending on soil type and land use). The soils selected for the study varied in terms of their typology, representing the most important Soil Reference Groups for temperate climates: Fluvisols, Regosols, Cambisols, Arenosols, Histosols and Luvisols. All the samples were collected within the uplands of Eastern-Central Poland, from rural and sub-urban areas. The typological diversity of the soils enabled the selection of diversified samples both in terms of the type of humus and the degree of its maturity. In addition, soil samples were collected from areas that were used for a variety of purposes (i.e., arable land, grassland, forest), which to a large extent are derivative of the soil production quality (Table 1). The collected soil samples were air-dried and passed through a 1 mm mesh sieve to DNA analysis. For analysis of HS, they were ground in a mortar and passed through a 0.25 mm sieve.

**Table 1:** Properties of soils used in experiments.

No.	Reference Soil Group (acc. to WRB)	Use	C_org_ g kg^−1^	C_HA_ g kg^−1^	C_FA_ g kg ^-1^	C_HA_/C_FA_	C_HS_ g kg^−1^	Texture class (WRB)	DNA concentration (ng/µl)	*A*_260_/*A*_280_^*^	*A*_260_/*A*_230_^*^
1	Fluvisol	Meadow	26.2	10.3	1.42	7.25	11.72	Loamy sand	25.5	1.93	1.19
2	Regosol	Plough field	11.2	2.8	0.91	3.08	3.71	Sandy loam	61.3	1.92	1.20
**3**	**Cambisol**	**Meadow**	**10.9**	**9.67**	**1.30**	**7.45**	**10.99**	**Loamy sand**	**11.7**	**1.98 (2.46)**	**0.44 (1.20)**
4	Cambisol	Plough field	7.7	5.99	1.02	5.87	7.01	Sandy loam	216.5	1.82	0.68
**5**	**Cambisol**	**Forest**	**20.1**	**14.97**	**1.92**	**7.8**	**16.89**	**Sandy loam**	**46.8**	**1.91 (2.07)**	**1.13 (1.4)**
**6**	**Arenosol**	**Forest**	**29.2**	**27.32**	**2.49**	**10.97**	**29.81**	**Sandy loam**	**32.5**	**1.89 (1.95)**	**1.31 (1.90)**
**7**	**Histosol**	**Meadow**	**40.3**	**34.66**	**2.70**	**12.84**	**37.36**	**Silt loam**	**200.8**	**1.92 (1.95)**	**1.49 (1.53)**
8	Luvisol	Plough field	9.3	3.36	0.91	3.69	4.27	Silt loam	30.1	1.85	1.20

**Notes:**

C_org_, organic carbon content; C_HA_, humic acids carbon content; C_FA_, fulvic acids carbon content; C_HA_/C_FA_, ratio of humic/fulvic acids carbon content; C_HS_, humic substances carbon content.

* Purity of DNA extracted from tested samples expressed as the ratio of absorbance *A*_260_/*A*_280_ and *A*_260_/*A*_230_ (values after additional purification of bold samples are presented in brackets) (Tables S1 and S2).

### Analysis of HS

Before extraction, the organic carbon content in studied soil samples (total organic carbon) was determined according to the spectrophotometric method described by Orłow and Grindel (Orłow, Grisina & Jerosicewa, 1969), and then the extraction of HS fractions was performed using the procedure recommended by the International HS Society (Swift, 1996) (Table 1; Tables S2 and S3).

The extraction was performed in the following steps: (1) decalcitation of soil samples with HCl, (2) triple extraction with NaOH (m/V = 1:10; time extraction = 4 h), (3) determination of carbon content in HS alkaline extracts using Orłow and Grindel’s spectrophotometric method (Orłow, Grisina & Jerosicewa, 1969), (4) HA precipitation by HCl, (5) determination of carbon content in FA non-purified solutions according to Orłow and Grindel’s spectrophotometric method (Orłow, Grisina & Jerosicewa, 1969), (6) HA purification by re-dissolve in KOH by adding solid KCl, (7) HA re-precipitation by HCl, (8) washing out by re-distilled water until the negative Cl^–^ test with silver nitrate AgNO_3_, (9) HA re-dilution in NaOH, and (10) determination of carbon content in HA solutions according to Orłow and Grindel’s spectrophotometric method (Orłow, Grisina & Jerosicewa, 1969).

### Spectral characteristics of HS fractions

In order to obtain the full characteristics of HA and FA extracts, spectrophotometric measurements in the UV–Vis range were performed with the SPECORD UV–Vis M-42, a computer-aided dual beam spectrophotometer with START software by Carl Zeiss Jena. All solutions subjected to photometric analysis were characterized by the same carbon concentration: 10 mgC·dm^−3^ in a solution of 0.05 moldm^−3^ NaHCO_3_. On the basis of the obtained absorption spectra, the following coefficients were calculated (Table 2; Table S3): *A*_465/665_, expressing the ratio of absorbance values at λ = 465 nm and λ = 665 nm (Chen, Senesi & Schnitzer, 1977); *A*_280/465_, representing the quotient of absorbance values at λ wavelengths: 250 nm or 280 nm and 465 nm (Gonet & Dębska, 1998); Δlog *K* = log *K*_400 nm_ − log *K*_600 nm_, where *K* is the absorbance value at 400 and 600 nm (Kumada, 1987); Δ*A*_1_/Δ*A*_2_, where: Δ*A*_1_ = *A*_290 nm_ − *A*_333 nm_, Δ*A*_2_ = *A*_357 nm_ − *A*_416 nm_ (Gołębiowska, 2004).

**Table 2:** Spectral characteristics of the material.

No.	Humic acids					Fulvic acids				
	*A*_250/465_	*A*_280/465_	*A*_465/665_	Δ*A*_1_/Δ*A*_2_	Δlog *K*	*A*_250/465_	*A*_280/465_	*A*_465/665_	Δ*A*_1_/Δ*A*_2_	Δlog *K*
1	7.08	5.96	4.36	1.46	0.68	16.82	12.44	5.69	1.82	0.89
2	7.94	6.85	5.46	1.53	0.76	17.35	12.57	5.46	2.06	0.87
3	7.65	6.47	4.82	1.56	0.70	17.63	12.98	7.10	1.83	0.97
4	6.68	5.60	4.70	1.56	0.67	24.3	16.92	5.00	2.12	0.95
5	7.38	5.92	5.03	1.75	0.67	19.88	14.26	7.19	1.80	1.01
6	7.77	6.29	5.09	1.62	0.70	20.21	14.36	7.16	1.72	1.04
7	6.88	5.76	4.05	1.44	0.65	12.88	9.75	5.65	1.56	0.84
8	9.03	7.73	5.41	1.53	0.80	19.48	14.03	4.98	1.82	0.94

**Notes:**

*A*_250/465_, the ratio of absorbance values at λ = 250 nm and λ = 465 nm; *A*_280/465_, the ratio of absorbance values at λ = 280 nm and λ = 465 nm; *A*_465_/_665_, the ratio of absorbance values at λ = 465 nm and λ = 665 nm; Δlog *K* = log *K*_400 nm_ − log *K*_600 nm_, where *K* is the absorbance value at 400 nm and 600 nm; Δ*A*_1_/Δ*A*_2_, where: Δ*A*_1_ = *A*_290 nm_ − *A*_333 nm_, Δ*A*_2_ = *A*_357 nm_ − *A*_416 nm_ (Table S3).

Additionally, fluorescence measurements (Fluorescence Spectrophotometer F-7000; Hitachi, Chiyoda, Japan) were performed on HA and FA extracts (Table 3; Table S4). The Fl emission spectra were recorded at four wavelengths of excitation light λ_ex_: 254 nm, 320 nm, 370 nm and 460 nm, at a same carbon concentration of 10 mg Cdm^−3^ in a solution of 0.05 moldm^−3^ NaHCO_3_. On the basis of Fl emission spectra, the following were determined: (i) the humification index (HIX defined as the integrated intensity of emission intensity in 435–480 nm divided by that in 300–345 nm at excitation 254 nm), characterizing the degree of maturity of soil humus (Zsolnay et al., 1998), (ii) the biological activity input index (BIX) calculated by emission intensity at 380 nm divided by 430 nm at λ_ex_ = 310 nm, which is an indicator of the relative share of recently produced fresh material (Huguet et al., 2009), (iii) the fluorescence index (*f*_450_/*f*_500_ calculated as a ratio at λ_ex_ 450 nm to that at λ_ex_ 500 nm, given that λ_ex_ = 370 nm). This strongly correlates with the degree of structure complexity and aromaticity (McKnight et al., 2001) and (iv) the *A*_465_ index, whose measure is the area under the fluorescence spectral curve λ_ex_ 465 nm. This parameter describes the progress of the humification process and the increase in the concentration of free radicals (Milori et al., 2002).

**Table 3:** Fluorescent characteristics of the material.

No.	Humic acids								Fulvic acids							
	HIX	BIX	*f*_450_/*f*_500_	*A*_465_	IF_460_/*a*_460_	IF_310_/*a*_310_	IF_370_/*a*_370_	IF_245_/*a*_245_	HIX	BIX	*f*_450_/*f*_500_	*A*_465_	IF_460_/*a*_460_	IF_310_/*a*_310_	IF_370_/*a*_370_	IF_245_/*a*_245_
1	3.36	0.88	1.20	421	5.69	7.87	6.52	3.26	7.30	0.88	1.64	433	2.10	7.55	6.23	3.96
2	4.85	0.54	1.22	501	7.11	8.43	7.52	3.46	8.51	0.46	1.63	356	2.37	9.99	7.69	5.81
3	4.72	0.34	1.23	385	5.66	7.91	6.44	3.47	9.52	0.54	1.64	392	1.92	8.42	6.34	4.38
4	6.95	0.59	1.15	644	7.54	9.30	7.29	3.60	9.19	0.49	1.75	372	3.36	15.93	12.02	8.18
5	5.80	0.59	1.16	530	6.79	10.37	7.84	4.02	9.00	0.50	1.64	370	1.84	9.75	6.39	4.72
6	5.66	0.56	1.19	515	8.24	11.90	9.12	5.23	8.36	0.40	1.60	381	2.11	9.55	6.26	5.16
7	9.74	0.58	1.12	367	4.47	5.97	5.01	2.40	10.00	0.41	1.61	456	1.39	5.54	4.18	3.03
8	4.36	0.54	1.28	438	8.42	10.24	9.35	4.24	7.87	0.57	1.69	297	3.37	14.09	11.15	8.53

**Note:**

HIX, humification index, BIX, biological index, *f*_450_/*f*_500_, fluorescence index, *A*_465_, humification index, IF_x_/*a*_x_, fluorescence efficiency index (Table S4).

### DNA experimental design according to the CTAB protocol

Soil samples (1g) in duplicate were suspended in 20 cm^3^ of extraction buffer (100 mM·dm^−3^ Tris–HCl (pH-8.2); 100 mM EDTA (pH-8); 1.5 M·dm^−3^ NaCl) and incubated at room temperature for 1 h with shaking at 150 rpm. Samples were re-extracted in 20 cm^3^ of the CTAB buffer (50 mM·dm^−3^ Tris-HCl, 20 mM·dm^−3^ EDTA, 150 mM·dm^−3^ NaCl, 27 mM·dm^−3^ CTAB pH-8). Supernatants were collected by low speed centrifugation at 2,097×*g* for 10 minutes (min). Next, 1 cm^3^ of lysis buffer (20% w/v SDS; lysozyme, 20 mg·cm^−3^; proteinase K, 10 mg·cm^−3^; N-lauroyl sarcosine, 10 mg·cm^−3^) was added to the supernatant followed by incubation at 37 °C for 1 h with vigorous shaking every 15 min. After incubation, 80 μl CTAB/NaCl (10% w/v, 70 mM·dm^−3^) was added to the mixture and incubated at 65 °C for 15 min. The samples were centrifuged at 8,385×*g* for 10 min at 4 °C. The upper aqueous phase was extracted with an equal volume of phenol:chloroform:isoamyl alcohol (P:C:I = 25:24:1) at 8,385×*g* for 20 min at 4 °C. The DNA was treated by adding a 10^−1^ volume of 7.5 mol·dm^−3^ potassium acetate and subsequently precipitated by adding two volumes of chilled absolute ethanol for 30 min at 4 °C. The DNA precipitates were collected by centrifugation at 6,708×*g* for 10 min, air-dried and suspended in 20 µl of sterile D/W. DNA extracts from soils containing a high concentration of HA were pooled to provide sufficient DNA for the purification experiments. We used the Wizard^®^ DNA Clean-Up System from Promega. After the isolation, the measurement of DNA concentration and purity was performed using a NanoDrop (SensoQuest Labcycler), measuring the absorption coefficients *A*_260_/*A*_280_ and *A*_260_/*A*_230_.

### Polymerase chain reaction amplification assay

In the next step, the material in the ratio of 100 ng·cm^−3^ was subjected to Polymerase chain reaction (PCR). The reaction mixture consisted of 12.5 μl of MM, 2.5 μl of the dye, nuclease-free water and 0.5 μl of the two primers: 806R (5′-GG ACTACHVGGGTWTCTAAT-3′) and 515F (5′-GTGCCAGCMGCCGCGGTAA-3′). The PCR was carried out under the following conditions: pre-denaturation at 95 °C for 5 min, denaturation at 95 °C for 1 min for 30 cycles, annealing at 58 °C for 1 min for 30 cycles, elongation at 72 °C for 0.5 min for 30 cycles and the final elongation at 72 °C for 7 min. The obtained material was subjected to electrophoresis, with a current of 5 V·cm^−1^ for 1.5 h in 1.4% agarose gel containing 5 μg·m^−3^ ethidium bromide.

## Results

In line with expectations, the studied soils varied in terms of the properties of HS (Table 1). The organic carbon content ranged from 7.7 g·kg^−1^ (Cambisol, Arable soil) to 40.3 g·kg^−1^ (Histosol, meadow). The direction of organic matter transformation, expressed by the ratio of HA carbon to FA carbon, was related to the type and land use of the soil. The lowest values were recorded in the cultivated field (Regosol and Luvisol), with the highest in the meadow (Histosol, meadow). The indices for HA and FA calculated on the basis of absorbance showed high variability within the studied material, which indicates a differentiated organic substance character (Table 2). Coefficient *A*_465_/_665_, negatively correlating with the size and weight of molecules, reached a relatively wide range of values—4.05–5.46 for HA and 4.98–7.19 for FA. The index value is dependent on the soil type and land use. Parameter *A*_250_/_465_ corresponds well to the degree of condensation of aromatic structure and molecular weight, and its lower values points to higher condensation and molecular weight.

Another parameter is the Δlog*K* coefficient introduced by Kumada (1987). The recorded values—from 6.68 to 9.03 (HA) and 12.88 to 24.3 (FA)—indicated these fractions in the studied soils had various levels of maturity. Some authors (Kowalczuk et al., 2010; Fong et al., 2006) also suggest that differences of absorption properties of humic acids are not significant enough to enable absorption parameters be used as indicators of the degree of humification.

The ΔA_1_/ΔA_2_ coefficient allows for an indirect, relative and comparative determination of the share of absorbing structures in the area characteristic of phenols and aromatic hydroxyacids; the values recorded—1.44–1.75 (HA) and 1.56–2.12 (FA)—indicated differences in the number of the above-mentioned structures in the studied samples.

The obtained fluorescence parameters also showed spatial variations in the properties of the studied soils. The HA and FA extracted from studied soils exhibit quite different optical properties. The tested HS fractions are characterized by different emission fluorescence yields (Table 3). Many factors, including the orientation of functional groups, molecular “elasticity” and/or molecular “crowding” affect the fluorescence emission ability. Various structural associations of OM molecules may differentiate their spectrophotometric properties and results in difficulties in the correlation with other parameters. Fluorescence and absorbance methods explain only a part of structure of those molecules. To assess the degree of the maturity of the soils of different origins, the HIX index, calculated from the fluorescence emission spectra, is also used (Zsolnay et al., 1998). The obtained HIX values (3.36–9.74 for HA and 7.3–10.0 for FA) indicate a variable degree of maturity of the organic matter of the studied soils. Higher HIX values correspond to more mature organic material and stronger aromaticity. Some authors (Kowalczuk et al., 2013; Singh, Dutta & Inamdar, 2014) suggest that, HIX is more statistically stable as the fluorescence method is more sensitive. The *f*_450_/*f*_500_ coefficient negatively correlates with the complexity of the structure and the aromaticity (McKnight et al., 2001; Chen et al., 2003). It is also used to differentiate between the origins of organic matter. Higher values of the *f*_450_/*f*_500_ fluorescence index are assumed for FA compared to HA. The *f*_450_/*f*_500_ fluorescence index values of the samples tested ranged from 1.12 to 1.28 for HA and 1.60 to 1.75 for FA, which reflects the different structural complexity of these substances. The *A*_465_ humification coefficient proposed by Milori et al. (2002) also provides information on the degree of the internal transformation of HS associated with the progress of humification processes. *A*_465_ index values of 297–644 were obtained, indicating the differentiation of organic matter transformation. Another parameter used to characterize the optical properties of HS is the BIX index. The highest BIX index values are determined for the HA and FA samples from Fluvisols (0.88), the lowest values for HA from Cambisol 3 (0.34). All the other samples are characterized by an intermediate biological activity (0.40–0.59) (Table 3).

The concentration and purity of DNA were analyzed for all soil group types (Table 1). The yields varied significantly between the eight different soil samples. A higher DNA concentration was obtained in the Cambisol arable soils samples: 216.5 ng·μl^−1^ compared to the Cambisol meadow: 11.7 ng·μl^−1^. The purity of the extracted DNA was evaluated by measuring the *A*_260/280_ and *A*_260/230_ ratios to indicate different contaminations. *A*_260/280_ is used to evaluate the presence of proteins and RNA. The optimal *A*_260/280_ ratio is between 1.8 and 2.0 (Glasel, 1995). The DNA ratio from all soil samples ranged between 1.82 and 1.92, indicating that protein and RNA contamination were negligible. *A*_260/230_ is used to evaluate the presence of aromatic compounds, such as phenol or benzene derivatives as part of the structure of HA. The optimal ratio is between 1.8 and 2.2 (Olson & Morrow, 2012). Our results show that the *A*_260/230_ ratios were lower than 1.8, which may indicate the presence of HA or phenol.

**Figure 1:**
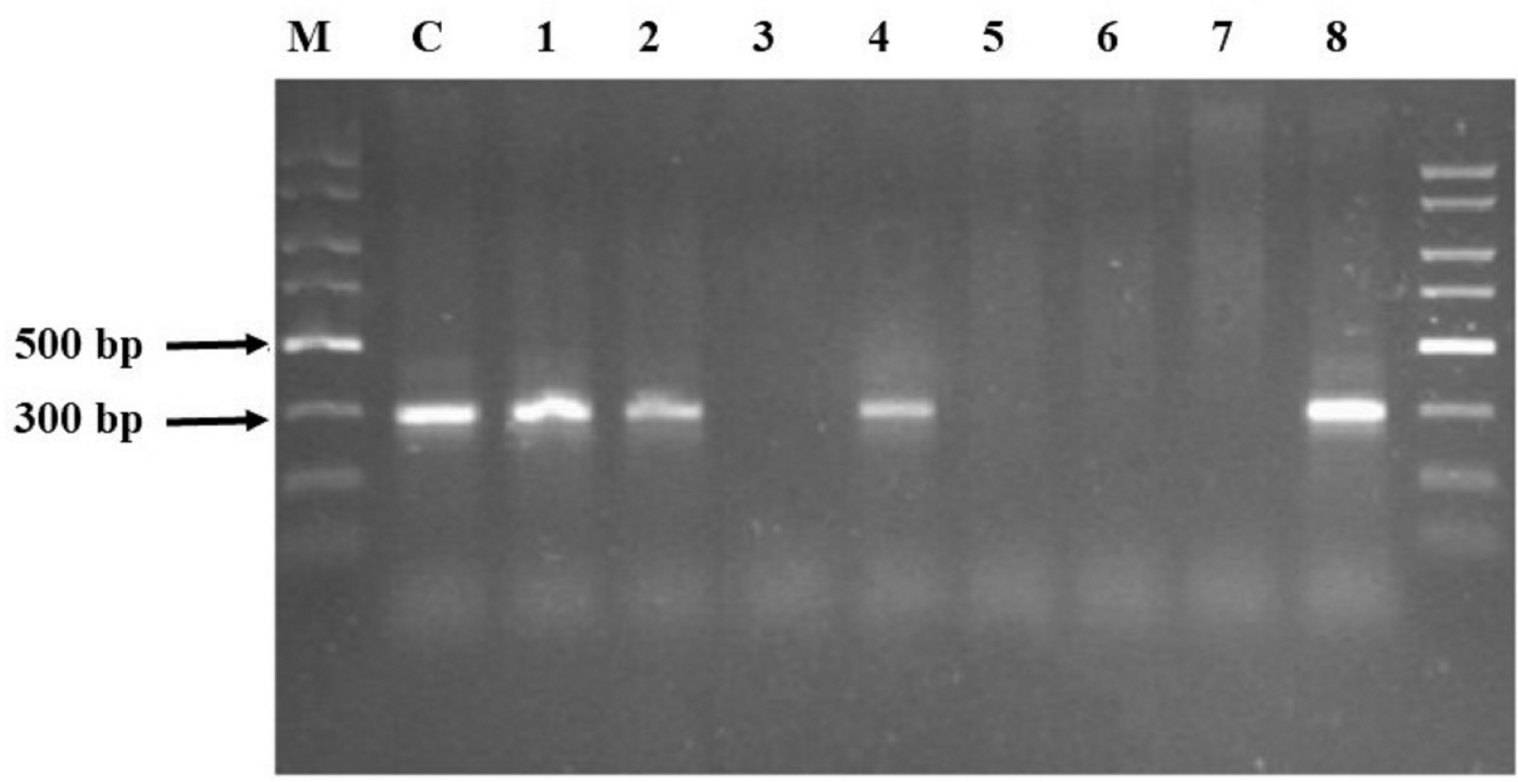
Agarose electrophoresis products obtained using the GeneMATRIX Soil DNA Purification Kit from EURx. The visible products were obtained in Samples 1, 2, 4 and 8. Lane C: pure DNA isolated from *E. coli* DH5 alpha used as positive control verifying the PCR reaction; Lane 1–8: soil samples; lane M: Perfect Plus 2kb DNA Lauder Marker.

**Figure 2:**
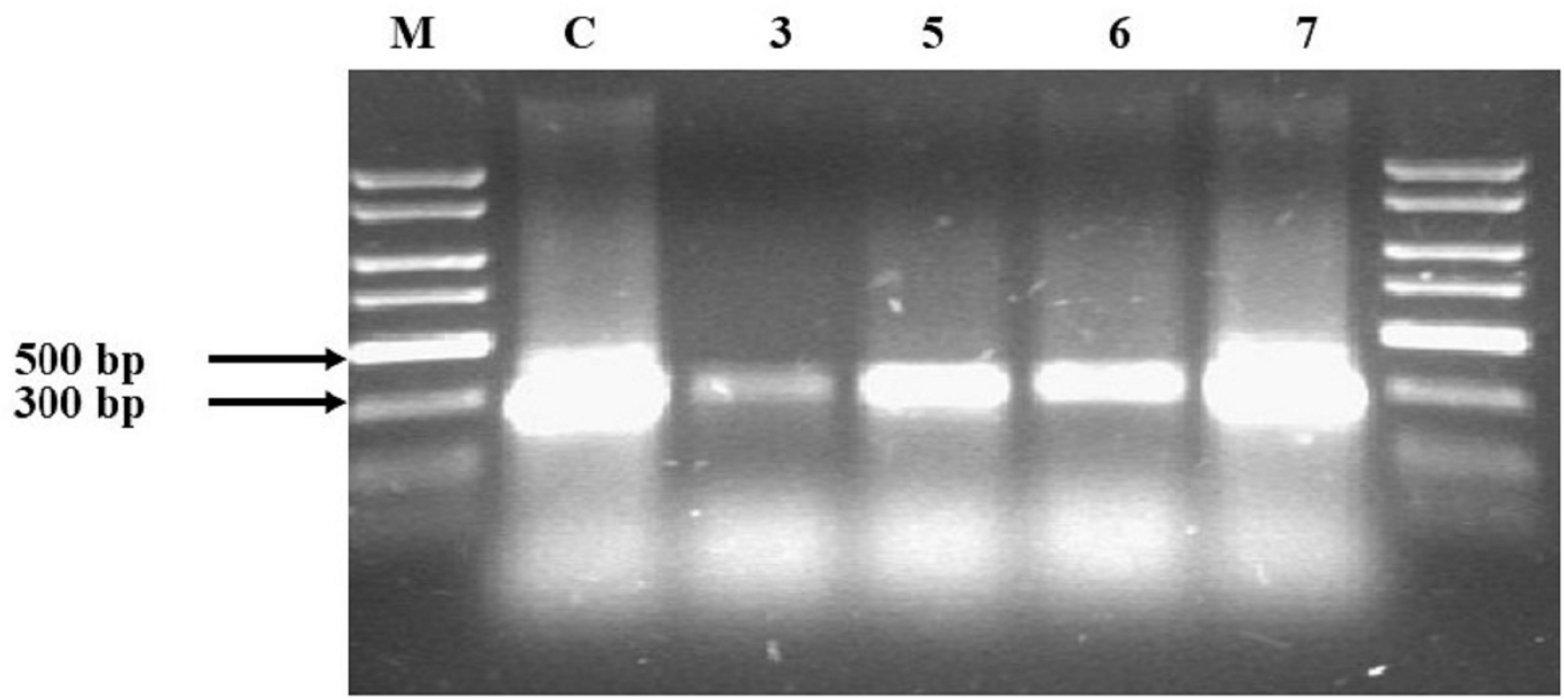
Agarose electrophoresis of Samples 3, 5, 6 and 7, which were not obtained during the first attempt. The samples were subjected to a clean-up procedure with the Wizard DNA Clean-Up System and re-used. Lane 3, 5, 6 and 7: soil samples; Lane C: pure DNA isolated from *E.coli* DH5 alpha used as positive control verifying the PCR reaction; lane M: Perfect Plus 2kb DNA Lauder Marker.

Agarose electrophoresis of the obtained PCR product gave the visible band size 300 bp only in half of the samples (Fig. 1). The samples where no product appeared—Samples 3, 5, 6 and 7—underwent a clean-up procedure due to the high concentration of HS. For this purpose, a Wizard^®^ DNA Clean-Up System was used. The purified material was re-used in the PCR reaction and the products were subjected to agarose electrophoresis. Following this procedure, the products that had previously not been visible appeared (Fig. 2). The resulting product was gene 16S rRNA.

The HS carbon content in Samples 3, 5, 6 and 7, mentioned above, was 10.99, 16.89, 29.81 and 37.36 g kg^−1^, respectively. This shows that the higher the HS carbon content in the soil, the poorer the DNA isolation result. The concentration of HS carbon was high (when compared to other samples) and the isolation of DNA was successful only in Sample 1 (Fluvisol, meadow); however, it is worth mentioning that only half of the total carbon in this sample was connected with HS.

## Discussion

It is known that different factors influence DNA yields: organic matter content (Zhou, Bruns & Tiedje, 1996; Miller et al., 1999; Sagova-Mareckova et al., 2008), total and biomass soil carbon (Lloyd-Jones & Hunter, 2001), particle size, water content (Burgmann et al., 2001; Pietramellara et al., 2009) and pH (Wolińska et al., 2012). The existence of such a number of different factors affecting DNA yields can be explained by their close correlation or similar mechanisms of influence.

Numerous studies have demonstrated the mechanisms of DNA binding to soil minerals or HS compounds (Pietramellara et al., 2009; Wolińska et al., 2012). The adsorption of DNA onto soil components impedes DNA degradation and constitutes a major mechanism of DNA molecule persistence in soil, a probable explanation for the ability of DNA to remain stable in the environment (Pietramellara et al., 2009; Wolińska et al., 2012).

In our study, DNA was isolated using the CTAB-based soil DNA extraction method and the obtained material was not pure. Many authors suggest an additional purification step to remove humic materials from DNA extracts (Young et al., 1993; Zhou, Bruns & Tiedje, 1996; Ranjard et al., 1998; Sharma, Capalash & Kaur, 2007). Our tests confirmed the need for an additional cleaning procedure for Samples 3, 5, 6 and 7. The basic premise for cleaning was the lack of amplification products (Fig. 1). The limits of factors *A*_260_/*A*_280_ and *A*_260_/*A*_230_, which determine the need for cleaning, are difficult to determine clearly, but all of these values increased after cleaning. The additional purification step also improved the removal of contaminants in studies by other authors (Zhou, Bruns & Tiedje, 1996; Li et al., 2011; Yamanouchi et al., 2018). However, given the lack of information on the optical properties of HS in those studies, it is difficult to make a clear reference to those results.

According to various researchers (Sagova-Mareckova et al., 2008; Técher et al., 2010) DNA contamination with HS has resulted in PCR inhibition and endonuclease reaction restriction. This suggests that during the DNA extraction process, not much attention is still paid to the very complex soil components such as low-molecular-weight organic acid (oxolic acid), cations (e.g., Mg^2+^, Ca^2+^ and Al^3+^) and anions (e.g., NO_3_^−^, Cl^−^). Korolev et al. (1999) indicated that divalent (Mg^2+^, Ca^2+^) cations are substantially more efficient DNA adsorption mediators than monovalent cations (Na^+^, K^+^). The above-mentioned components could be co-extracted with DNA as well as interacting with DNA, consequently influencing DNA isolation from soil. Accordingly, it is reasonable to include a lysis buffer containing EDTA, which removes HS in addition to other compounds (He, Xu & Hughes, 2005).

Our results showed that the higher the HS carbon content in soil, the poorer the DNA isolation result. Different results were obtained in Tsai and Olson’s work (Tsai & Olson, 1992), who demonstrated that irrespective of the concentration of DNA in the reaction mixture, even a small amount of HS is able to inhibit the activity of Taq polymerase. It is worth mentioning that in the case of Fluvisol, high levels of HS carbon were detected, however, this constituted only half of the total carbon in this sample. Having noticed the relation between the HS carbon content and the effectiveness of DNA isolation, an attempt was made to determine which properties of their fractions influence this process. Despite the wide range of HA and FA parameters, no significant correlations were found in either the context of the spectral properties of individual extracts or the fluorescence intensity.

The type and the use of the soil are also not without significance. According to our results, it can be seen that whatever the type of soil used, the DNA fragment was obtained without the need for a clean-up procedure in all samples obtained from the land used as arable soils. In the case of soil from the land used as meadows, the DNA fragment was obtained in only one out of three samples. This also allows us to conclude that land use may be important factor in terms of the inhibition of Taq polymerase in DNA amplification, mainly because of the specific share of HS carbon in the total carbon pool.

## Conclusion

A direct relationship between land use (forest, meadow, plow field) and isolation of DNA was not observed. Soil type (Cambisol, Fluvisol, Regosol, Arenosol, Histosols, Luvisol) does not influence the effects of the procedure. No relationship between HS molecular size as well as structure and the DNA yield activity of Taq polymerase was found. The carbon content of HS was decisive in terms of the effectiveness of DNA isolation from soil samples at a value above 10 g kg^−1^. At the same time, an overwhelming share of the total pool of organic carbon samples needed additional purification.

## Supplemental Information

10.7717/peerj.9378/supp-1 Supplemental Information 1 Organic carbon content in analyzed soils.

10.7717/peerj.9378/supp-2 Supplemental Information 2 Carbon content of humic and fulvic acids.

10.7717/peerj.9378/supp-3 Supplemental Information 3 Spectral characteristics of humic and fulvic acids.

10.7717/peerj.9378/supp-4 Supplemental Information 4 Fluorescent characteristics.

## Additional Information and Declarations

### Data Availability

The following information was supplied regarding data availability: The data are available as Supplemental Files.
